# Systemic redox imbalance correlates with clinical aggressiveness and poor prognosis in canine inflammatory mammary carcinoma

**DOI:** 10.3389/fvets.2026.1847143

**Published:** 2026-06-02

**Authors:** Thanielle Novaes Fontes, Anna Hielm-Björkman, Carlos Humberto da Costa Vieira-Filho, Vitor de Moraes Pina de Carvalho, Laís Pereira Silva, Viviane Abreu Pedreira de Oliveira, Gisele André Baptista Canuto, Hanna Carvalho de Sá, Fernanda Barthelson Carvalho de Moura, Stella Maria Barrouin-Melo, Carlos Eduardo Fonseca-Alves, Alessandra Estrela-Lima

**Affiliations:** 1 Research Center on Mammary Oncology (NPqOM), Veterinary Medicine Hospital (HOSPMEV), Federal University of Bahia, Salvador, Brazil; 2 DogRisk Research Group, Department of Equine and Small Animal Medicine, Faculty of Veterinary Medicine, University of Helsinki, Helsinki, Finland; 3 Department of Analytical Chemistry of the Institute of Chemistry, Federal University of Bahia, Salvador, Brazil; 4 Department of Veterinary Surgery, University of São Paulo (USP), São Paulo, Brazil; 5 Department of Anatomy, Pathology and Veterinary Clinics, School of Veterinary Medicine and Animal Science, Federal University of Bahia, Salvador, Bahia, Brazil

**Keywords:** canine mammary tumors, comparative oncology, lipid peroxidation, malondialdehyde, prognostic biomarkers, redox status, total antioxidant capacity

## Abstract

**Introduction:**

Canine inflammatory mammary carcinoma (IMC) is a highly aggressive neoplasm characterized by rapid clinical progression, high metastatic potential, and poor prognosis. Increasing evidence suggests that systemic redox imbalance, resulting from a disproportion between reactive oxygen species and antioxidant defenses, plays a key role in tumor progression. This study evaluated serum total antioxidant capacity (TAC) and malondialdehyde (MDA) as systemic biomarkers of redox status in female dogs with IMC and non-inflammatory mammary tumors (NIMTs).

**Methods:**

Serum TAC and MDA levels were measured in 49 female dogs aged 8–18 years. The cohort included dogs with IMC (*n* = 20) and dogs with NIMTs (*n* = 29), the latter subdivided into aggressive tumors (*n* = 11) and low-grade tumors (*n* = 18). Biomarker levels were compared among groups and correlated with clinical aggressiveness and overall survival.

**Results:**

Systemic redox imbalance was significantly more pronounced in dogs with IMC. Serum MDA levels were markedly higher in the IMC group (3558.9 nmol/mL) than in dogs with aggressive (53.3 nmol/mL) or low-grade tumors (48.3 nmol/mL) (*p* < 0.001). In contrast, TAC levels were significantly lower in the IMC group (9.8 mmol/L) compared with the aggressive (14.0 mmol/L) and low-grade groups (12.6 mmol/L) (*p* < 0.001). Survival analysis confirmed the extremely poor prognosis associated with IMC, with a median survival of 16 days and 100% mortality, whereas 94.4% of dogs with low-grade tumors survived until the study endpoint (698 days). Higher MDA levels were strongly associated with shorter survival and greater clinical aggressiveness in IMC cases (p < 0.001).

**Discussion:**

These findings demonstrate that systemic redox imbalance is closely associated with the rapid progression and poor survival observed in canine IMC. Serum TAC and MDA may serve as valuable prognostic biomarkers and support the relevance of oxidative stress as a potential therapeutic target in aggressive mammary neoplasms. These results may also have comparative implications for human oncology.

## 1 Introduction

Breast cancer represents almost one in four cancer cases and one in six cancer deaths in women worldwide (1). The similarity between human and canine mammary tumors has consolidated the female dog as a translational model for oncological studies (2, 3). Among the tumor subtypes, inflammatory mammary carcinoma (IMC) stands out as a rare and highly aggressive form, associated with rapid progression, a high rate of metastasis, and lower survival rates in both humans and animals (4, 5).

Research in women with mammary cancer (MC) has demonstrated that reactive oxygen species (ROS) play a critical role in the etiology and progression of the tumor, even before its clinical onset (6, 7). Understanding the role of ROS in the pathophysiology of MC is crucial for developing therapeutic strategies that modulate oxidative stress (6, 7). Studies have described the mechanisms by which oxidative stress participates in the development of various types of cancer (8, 9). Authors have shown that an imbalance between ROS production and cellular antioxidant capacity can lead to genetic alterations, disrupt glycolytic activity, or affect epigenetics in the tumor microenvironment, thereby promoting carcinogenesis and tumor aggressiveness (10, 11).

The total antioxidant capacity (TAC) is a valuable indicator of a patient’s clinical status and guides therapies in cases of antioxidant dysregulation (12). The formation of malondialdehyde (MDA), a byproduct of lipid hydroperoxide decomposition, is used to estimate lipid peroxidation in biological systems (13).

In women with MC, higher MDA levels are associated with excessive ROS production and antioxidant deficiency (7). Recent studies on female dogs with MC have shown that ozone therapy increased TAC and reduced MDA, thereby improving the oxidative profile (14). An improvement in the oxidative profile not only reflected a greater antioxidant capacity in ozone-treated dogs but also had a positive impact on their response to treatment and quality of life (15).

As the regulation of redox homeostasis is vital for cellular health, understanding it with open possibilities to managing IMC (6, 16). To contribute to the knowledge base for the potential design of future therapies for canine IMC, we aimed to compare TAC and MDA levels in the blood of female dogs with IMC and other malignant tumors, and to correlate these levels with survival.

## 2 Materials and methods

### 2.1 Ethical aspects

All experimental procedures were performed in accordance with the guidelines established by the National Council for the Control of Animal Experimentation (CONCEA) of Brazil. This study was approved by the Research Ethics Committee on Animal Experimentation of the Federal University of Bahia (CEUA n° 01/21).

### 2.2 Animals

This study evaluated 150 female dogs of various breeds and ages diagnosed with mammary neoplasia, assisted by the Breast Oncology Research Center of the Federal University of Bahia, Brazil. However, only 49 dogs were selected to compose the experimental groups, recruited according to the inclusion and exclusion criteria.

Inclusion criteria were any breed, size, and age, presenting inflammatory carcinoma and other malignant mammary neoplasms, without a history of comorbidities (infectious/autoimmune diseases) or other types of non-mammary tumors. Exclusion criteria were detection of other tumor types or comorbidities during the study; owners’ failure to follow the schedule for bringing their dogs in for the clinical evaluations and sample collections; previous chemotherapy; and/or previous integrative treatments (ozone therapy, cannabis, and homeopathy).

Following clinical evaluation, the dogs were initially divided into two experimental groups: the inflammatory mammary carcinoma (IMC) group (*n* = 20), composed of dogs with a clinicopathological diagnosis of inflammatory carcinoma; and the non-inflammatory malignant mammary tumor (NIMT) group (*n* = 29), composed of dogs with operable tumors. Dogs in the NIMT group were further subdivided, based on histopathological analysis, into low-grade tumors (LgT; *n* = 18) and aggressive tumors (AT; *n* = 11). Aggressive tumors included those classified into the histopathological grade II/III and malignant adenomyoepithelioma.

### 2.3 Clinical evaluation and mastectomy

All dogs underwent a comprehensive clinical evaluation, including a detailed clinical history, a review of reproductive records, and a physical examination. Following this, blood was collected for assessment of biochemical and hematological parameters. A thoracic radiograph was performed in three views, and an abdominal ultrasound was performed to identify metastases.

Clinical staging was based on tumor size (T), regional lymph node involvement (N), and the presence or absence of distant metastases (M), following the TNM classification system (17, 18). Macroscopic evaluation of inguinal and axillary lymph nodes was performed by palpation. Dogs with NIMT underwent unilateral mastectomy with the removal of regional lymph nodes. Neoplastic involvement of lymph nodes was confirmed by histopathological examination after mastectomy of dogs with NIMT or after death of dogs with IMC.

The specific survival (SS) was defined as the period (in days) between mastectomy and death for each animal caused by disease progression, and the overall survival time was defined as the time (in days) between the surgical excision of the primary tumor and the date of death (by any reason) or until the end date of the study. The follow-up was maintained until the end of the study or the animal’s death.

### 2.4 Histopathological classification and grading

Immediately after surgery of the NIMT-dogs or after death of IMC-dogs, the removed mammary chain and regional lymph nodes were fixed in 10% neutral formalin buffered with 10% phosphate (monobasic and dibasic). At first, histopathological examination and tumor characterization were performed to determine the tumor diagnosis and select cases for the study. The histological sections of the selected mammary neoplasms were re-evaluated under light microscopy. Whenever necessary, new histological processing of the reserve samples was performed using the routine paraffin-embedding technique (19) with 4 μm histological sections stained with Hematoxylin–Eosin (HE) and analyzed under a conventional light microscope.

In all cases, two veterinary pathologists analyzed duplicate slides. The histological classification followed the criteria proposed by Zapulli et al. (20) and Cassali et al. (17). Histopathological grading was performed according to the Nottingham histological grading system (21), which assesses the percentage of tubular differentiation, evaluates nuclear pleomorphism, and determines the mitotic index. Dogs that received a diagnosis of IMC, of histopathological malignant tumor with grade II or III, aggressive, and nodal metastasis were subsequently referred for chemotherapy treatment.

### 2.5 Sample collection for oxidative profile

For oxidative profile analysis using malondialdehyde (MDA) and total antioxidant capacity (TAC) determination, blood (serum) samples were collected from female dogs with IMC on the day of the first consultation, and from dogs with NIMT on the day of surgery. Five milliliters (mL) of each blood sample were centrifuged at 3,000 x g for 10 min, and serum aliquots were separated, placed in Eppendorf tubes, and stored at −80 °C until laboratory processing.

#### 2.5.1 Total antioxidant capacity (TAC)

Trolox is a water-soluble analog of vitamin E widely used as an antioxidant standard. A Trolox-equivalent antioxidant capacity assay was used to determine the total antioxidant capacity (TAC), as described by Erel (22). Briefly, 2,2′-azino-bis (3-ethylbenzothiazoline-6-sulfonic acid) (ABTS) was generated by the reaction between ABTS diammonium salt (7.0 mmol L^−1^) and potassium persulfate (2.45 mmol L^−1^) and kept in the dark at room temperature for 16 h. The ABTS solution was subsequently diluted with phosphate-buffered saline (PBS) until the absorbance at 734 nm reached 0.70 ± 0.05. The TAC determination was performed using a spectrophotometer (VersaMax™ Microplate Reader, Thermo Fisher Scientific, Waltham, United States) at 734 nm, after a 6-min reaction between 198 μL of ABTS diluted in PBS and 2 μL of standard solutions, samples, or blank. Ethanolic solutions of 6-hydroxy-2,5,7,8-tetramethylchroman-2-carboxylic acid (Trolox), in the range of 0.25 to 2.00 mmol L^−1^, were used for triplicate calibration. PBS was used as the blank to correct the absorbance readings. The TAC was calculated based on the decrease in absorbance and expressed relative to the Trolox standard (mmol L^−1^).

#### 2.5.2 Malondialdehyde (MDA)

Serum MDA concentrations were determined as described by Tang et al. (23). MDA was produced by reacting 1:100 (v/v) bis (dimethyl acetal) malondialdehyde with 1% (v/v) sulfuric acid, maintained at room temperature for 2 h. The solution was subsequently diluted 10-fold with 1% (v/v) sulfuric acid solution. The calibration curve was prepared from a stock solution of MDA (165 mmol/L) in triplicate over the concentration range of 0.013–6.63 nmol. Samples were prepared by mixing 20 μL of serum samples with 500 μL of sulfuric acid (42 mmol L-1) and adding 125 μL of phosphotungstic acid. The mixture was homogenized by vortexing, incubated at room temperature for 5 min, and then centrifuged (15,000 × g for 5 min). 102 μL of butylated hydroxytoluene (BHT) (2.5 mmol L^−1^) on ice and 98 μL of deionized water were added to the sediment. Two hundred microliters of samples and standard solutions were mixed with 600 μL of thiobarbituric acid (TBA) (prepared in glacial acetic acid). The mixture was then incubated at 95 °C for 1 hour, followed by an ice bath for 10 min. Absorbance was measured at 532 nm using a spectrophotometer (VersaMax™ Microplate Reader, Thermo Fisher Scientific, Waltham, United States). The total MDA concentration was expressed in nanomoles per milliliter.

### 2.6 Statistical analyses

Data were tabulated for descriptive and graphical analyses. The choice between parametric and non-parametric tests was based on the Kolmogorov–Smirnov test for normality. For normally distributed variables, analysis of variance (ANOVA) followed by Tukey’s *post hoc* test was used to compare group means. Categorical variables were compared using Fisher’s exact test. Survival curves were estimated using the Kaplan–Meier method, and differences between groups were assessed with the log-rank test. Spearman’s correlation analysis was used to evaluate associations between the studied variables and survival. Multivariate survival analysis was performed using Cox proportional hazards regression. For TAC and MDA, cut-off values were defined based on the sample means, and cases were categorized into groups above or below each mean.

Multiple correlation analyses were conducted to assess the relationships among clinical variables, histopathological grade, overall survival, and oxidative stress biomarkers. The following variables were included: age, spaying status, tumor histological grade, overall survival time, serum TAC, and MDA concentrations. Pairwise correlations were computed using Pearson’s correlation coefficient (r). The univariate analysis evaluated the isolated association of each variable with survival, whereas the multivariate Cox model assessed the independent effect of these variables after mutual adjustment. In parallel, the correlation matrix was used as an exploratory approach to visualize pairwise relationships among clinical, pathological, and oxidative variables and therefore does not replace the multivariate survival model.

The strength of correlations was classified according to the absolute value of the correlation coefficient (|*r*|), as follows: weak (|*r*| = 0.20–0.39), moderate (|*r*| = 0.40–0.59), and strong (|*r*| = 0.60–0.79); values ≥ 0.80 were considered very strong. Correlation coefficients were displayed in a correlation matrix (heatmap), with positive and negative associations represented by a diverging color scale, and color intensity reflected correlation magnitude. The significance level adopted was *p* < 0.05, corresponding to a 95% confidence interval, with a two-tailed analysis. Analyses were performed using statistical programs SPSS® 26.0 for Windows and GraphPad Prism 8.0.2.

## 3 Results

### 3.1 Clinical and pathological features

The dogs’ ages in this study ranged from 8 to 18 years, with a mean age of 11.8 ± 0.5 years. There was a predominance of mixed-breed dogs across all groups (18/49; 36.7%), followed by Poodle dogs (9/49; 18.4%). The Maltese, Pinscher, and Pitbull breeds also accounted for 6.1% each (3/49). Regarding reproductive status, 85.7% (42/49) of the dogs were neutered (Table 1).

**Table 1:** Comparison of TAC and MDA values, epidemiology, and prognostic factors.

Clinicopathological variables	IMC *n* = 20	Aggressive tumors *n* = 11	Low-grade tumors *n* = 18	Total *n* = 49	*p*-value
	Mean (±SD)	Mean (±SD)	Mean (±SD)	Mean (±SD)	
Age (years)	11,8 (±0,5)^A^	10,3 (±0,8)^A^	9,9 (±0,5)^B^	10,8 (±0,3)	0,010*
TAC (serum)	9,8 (±0,5)^A^	14,0 (±0,9)^B^	12,6 (±1,0)^B^	11,7 (±0,5)	<0,001*
MDA (serum)	3,558,9 (±342,8)^A^	53,3 (±15,1)^B^	48,3 (22,7)^B^	1,531,8 (±297,7)	<0,001*
Survival (days)	25,1 (±5,5)^A^	326,3 (±58,3)^B^	489,1 (±35,6)^C^	253,4 (±35,7)	<0,001*
Histopathological grade					<0.001*
I	0	0	14 (100%)	14 (51.9%)	
II	1 (20%)	6 (75%)	0	7 (25.9%)	
III	4 (80%)	2 (25%)	0	6 (22.2%)	
Breed					0.411
Bassethound	0	0	1 (5.6%)	1 (2%)	
Cocker spaniel	0	0	1 (5.6%)	1 (2%)	
Daschund	1 (5%)	1 (9.1%)	0	2 (4.1%)	
Labrador	1 (5%)	0	1 (5.6%)	2 (4.1%)	
Maltese	2 (10%)	1 (9.1%)	0	3 (6.1%)	
Pinscher	0	1 (9.1%)	2 (11.1%)	3 (6.1%)	
Pitbull	1 (5%)	2 (18.2%)	0	3 (6.1%)	
Poodle	3 (15%)	3 (27.3%)	3 (16.7%)	9 (18.4%)	
Rottweiler	1 (5%)	0	1 (5.6%)	2 (4.1%)	
Schnauzer	0	0	1 (5.6%)	1 (2%)	
Shih Tzu	0	1 (9.1%)	0	1 (2%)	
Mixed-breed	11 (55%)	1 (9.1%)	6 (33.3%)	1 (2%)	
Yorkshire	0	1 (9.1%)	1 (5.6%)	1 (2%)	
Survival (condition)					<0.001*
Alive	0	4 (36.4%)	17 (94.4%)	18 (36.7%)	
Dead	20 (100%)	7 (63.6%)	1 (5.6%)	2 (4.1%)	

^A, B, C^Different superscript letters within the same row indicate significant differences between groups (*p* < 0.05, based on Tukey’s post-hoc test). *Significant differences (*p* < 0.005).

The LgT group consisted of 14 grade I mixed tumor carcinomas, three microinvasive mixed tumor carcinomas, and one microinvasive papillary carcinoma. The AT group had six grade II mixed tumor carcinomas, two grade III mixed tumor carcinomas, and three malignant adenomyoepitheliomas. Mixed tumor carcinoma, regardless of grade, was the most prevalent histological type of neoplasm in this study, corresponding to 69% of all cases. Micropapillary carcinomas were the most frequent histological type in tumors underlying the clinical presentation of IMC in this study, accounting for 75% of the cases in the IMC group.

### 3.2 Oxidative profile: total antioxidant capacity (TAC) and malondialdehyde (MDA)

**Figure 1:**
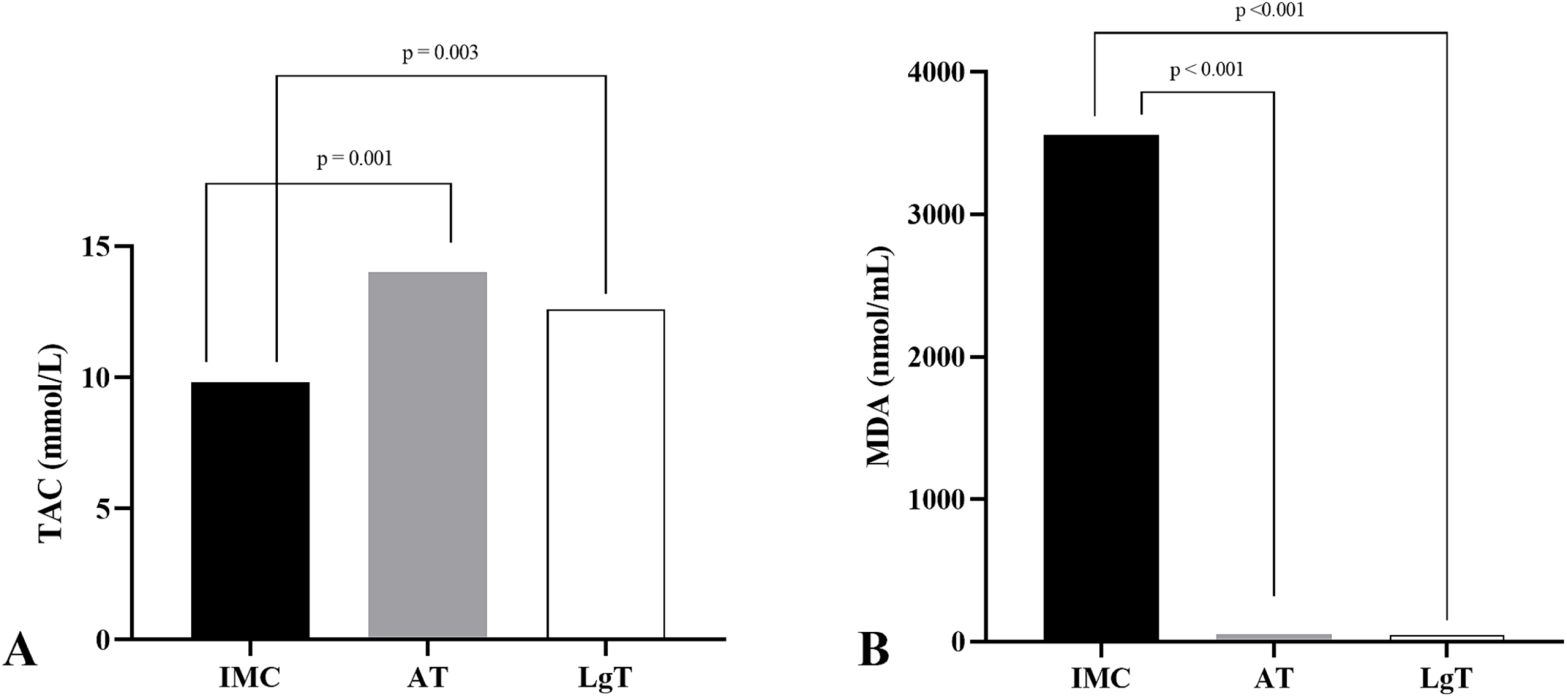
Serum levels of total antioxidant capacity (TAC) and malondialdehyde (MDA) in canine mammary tumors. **(A)** Comparison of serum TAC levels among the inflammatory mammary carcinoma (IMC) group, aggressive tumor (AT) group, and low-grade tumor (LgT) group. **(B)** Comparison of serum MDA levels among the inflammatory mammary carcinoma (IMC) group, aggressive tumor (AT) group, and low-grade tumor (LgT) group.

The mean serum TAC values were 9.8 mmol/L in the IMC group, 14.0 mmol/L in the AT group, and 12.6 mmol/L in the LgT group. The serum TAC values of the IMC group were significantly lower than those of the AT group, and subsequently those of the LgT group (*p* < 0.001) (Table 1; Figure 1A).

Differently, the mean serum MDA levels were higher in the IMC group (3558.9 nmoL/mL) as compared with those of the AT group (53.3 nmoL/mL), and those of the LgT group (48.3 nmoL/mL) (*p* < 0.001) (Table 1; Figure 1B).

Therefore, the results demonstrated significant differences in TAC and MDA values between the IMC, AT, and LgT groups, suggesting an association between patients with inflammatory carcinoma and elevated oxidative stress.

### 3.3 Comparison of survival curves

The minimum survival time was 3 days, observed in a dog from the IMC group. The maximum survival time was 698 days for a dog with a grade I mixed tumor carcinoma from the LgT group, which was alive at the time of study completion.

The IMC dogs achieved a maximum survival of 77 days, with a median of 16 days, and 100% mortality during the follow-up period. Dogs with aggressive tumors showed a median survival of 286 days and a death rate of 63.6%.

**Figure 2:**
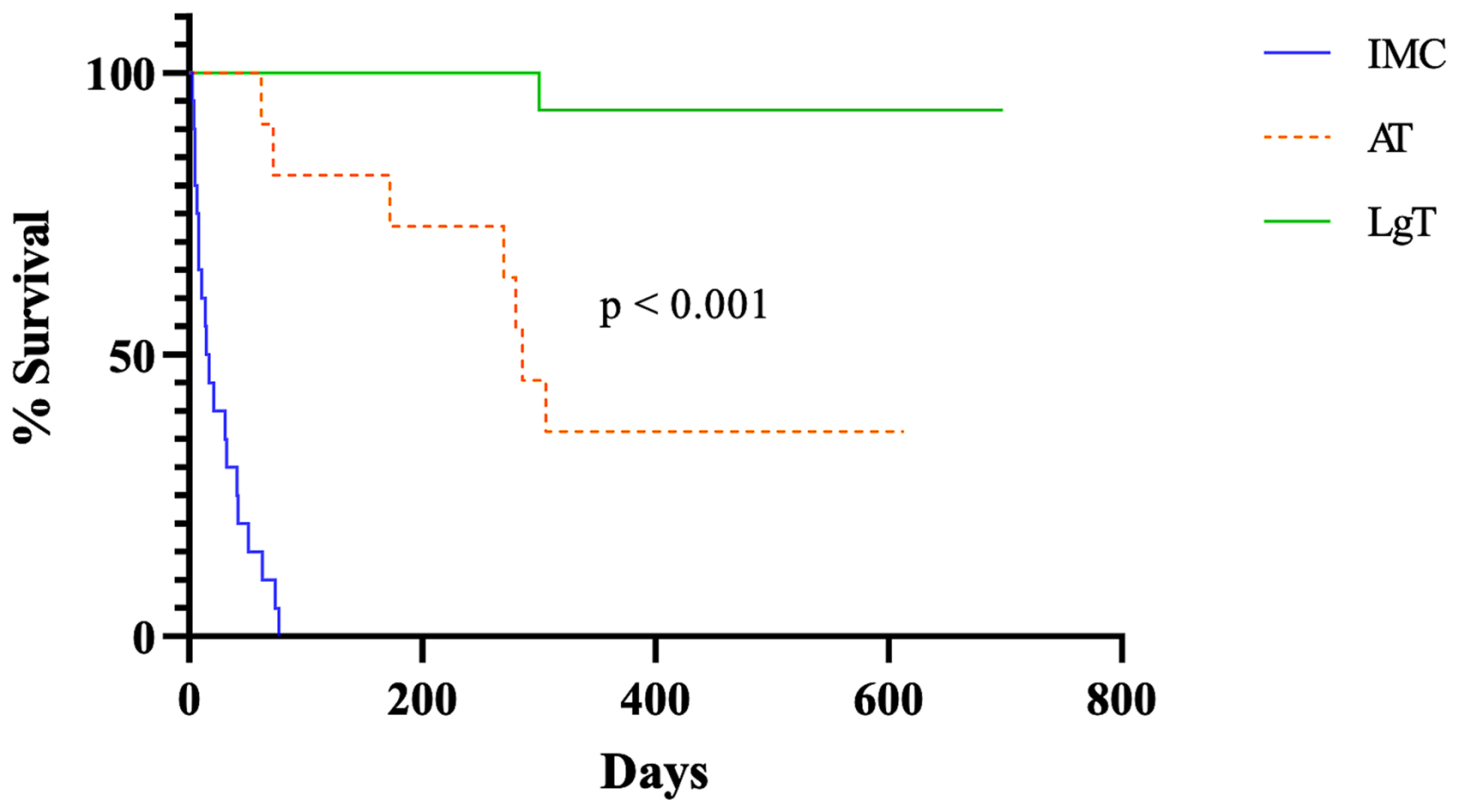
Kaplan–Meier survival curves of female dogs from the inflammatory mammary carcinoma (IMC), low-grade tumors (LgT), and aggressive tumors (AT).

In contrast, the low-grade tumor group showed a more favorable survival rate, with only one death and 94.4% of the dogs still alive at the end of the follow-up. The follow-up lasted 3 years. This was expected due to the histological type evaluated. The comparison between the groups revealed significantly higher survival rates in the low-grade tumor group than in the IMC group (*p* < 0.001) (Figure 2).

**Figure 3:**
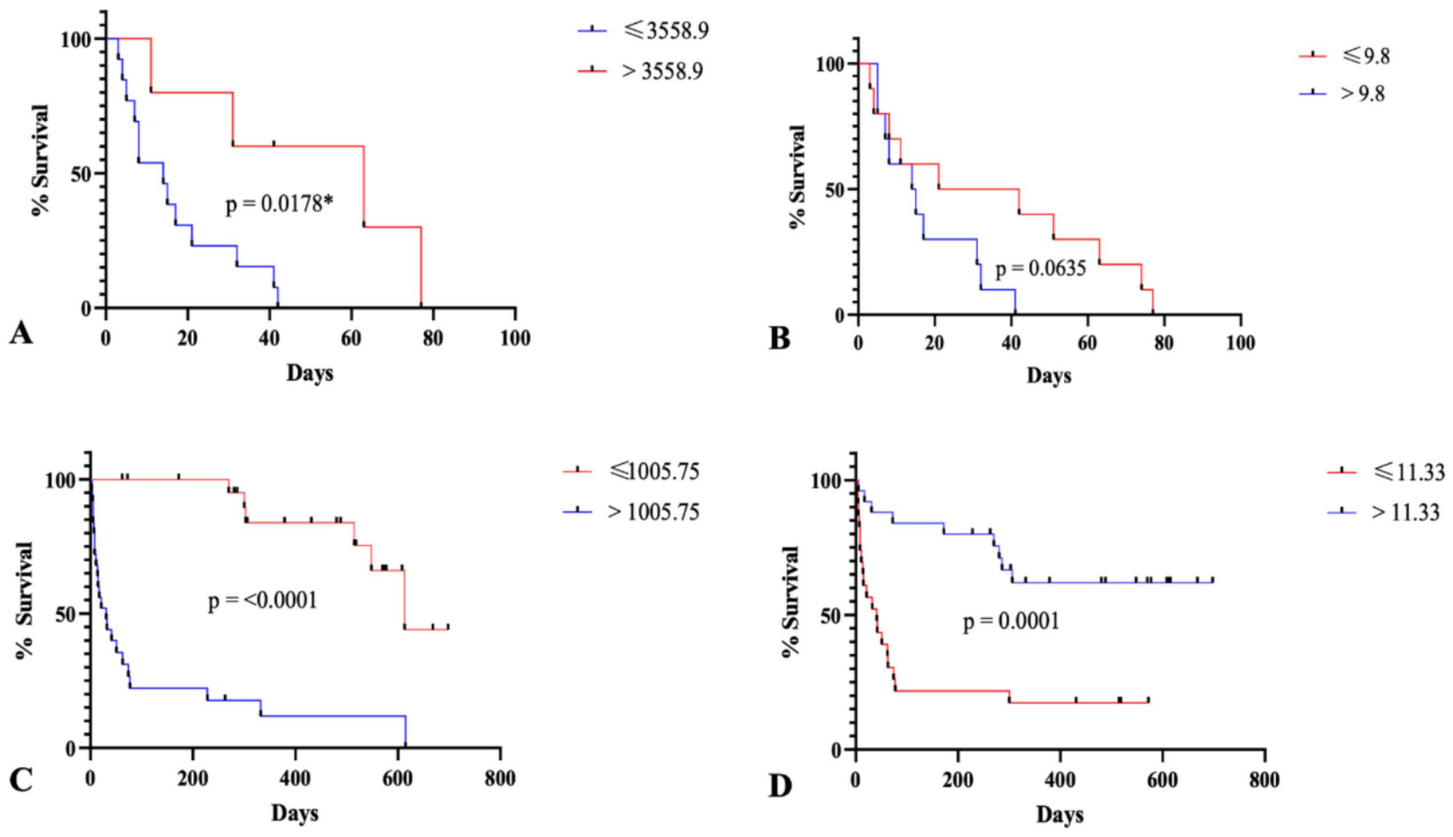
Kaplan–Meier survival curves for MDA and TAC concentrations, in the evaluated female dogs: **(A)** MDA of female dogs with inflammatory carcinoma (IMC); **(B)** TAC of female dogs with inflammatory carcinoma (IMC); **(C)** MDA of all female dogs in the study; **(D)** TAC of all female dogs in the study.

Global survival analysis was performed using Kaplan–Meier curves to evaluate the impact of MDA and TAC levels on overall survival. In the IMC group, stratified by an MDA cut-off of 3030.15 nmol/mL (Figure 3A), patients with MDA levels > 3030.15 nmol/mL had significantly lower survival than those with MDA ≤ 3030.15 nmol/mL (*p* = 0.0178). The maximum follow-up duration in this subgroup was approximately 80 days, reflecting the rapid progression of the disease.

In contrast, within the IMC group, with a TAC cut-off of 9.8 mmol/L (Figure 3B), no statistically significant difference in survival was identified between canine patients with levels above or below the cut-off (*p* = 0.0635). The general analysis of all mammary neoplasms, stratified by an MDA cut-off of 1005.75 nmol/mL (Figure 3C), showed that patients with MDA levels ≤ 1005.75 nmol/mL exhibited significantly longer survival than those with MDA levels > 1005.75 nmol/mL (*p* < 0.0001). Concurrently, in the general cohort stratified by a TAC cut-off of 11.33 mmol/L (Figure 3D), canine patients with TAC levels > 11.33 mmol/L had significantly longer survival than those with levels ≤ 11.33 mmol/L (*p* = 0.0001). The maximum follow-up duration for Figures 3C,D was approximately 700 days, reflecting the inclusion of a broader range of tumors with varied prognoses.

### 3.4 Univariate and multivariate analysis

Univariate analysis demonstrated a strong inverse association between tumor histological grade and canine survival, indicating that higher-grade tumors were associated with shorter survival times (*r* = −0.722; *p* < 0.001). In addition, MDA levels exhibited a moderate negative correlation with survival, suggesting that increased oxidative stress may be linked to poorer clinical outcomes (*r* = −0.427; *p* = 0.037) (Table 2). In the multivariate analysis, age, TAC levels, and MDA did not show significant associations with survival. In contrast, tumor grading was associated with a negative impact on canine patient survival (95% CI: 1.48–15.53; *p* = 0.009) (Table 2).

**Table 2:** Univariate and multivariate analysis of evaluated parameters related to survival.

Parameters	Univariate^a^		Multivariate^b^	
	Sperman’s Rho (CI 95%)	*p*-value	Hazard ratio (CI 95%)	*p*-value
Histopathological grade	−0.722 (−0.441– −0.911)	<0.001*	4.79 (1.48–15.53)	0.009*
Age	−0.099 (−0.336 – −0.481)	0.646	0.61 (0.35–1.07)	0.084
TAC (serum)	−0.391 (−0,737 – −0,012)	0.059	1.09 (0.81–1.47)	0.559
MDA (serum)	−0.427 (−0.006 – −0.742)	0.037*	1,1 (0.995–1.3)	0.307

^a^Sperman correlation; ^b^Cox regression; *Significant difference (*p* < 0.005).

The multiple correlation analysis revealed distinct associations among clinical variables, histopathological grade, survival, and oxidative stress biomarkers. Overall survival showed a moderate negative correlation with histopathological grade (*r* = −0.55), indicating reduced survival in dogs with higher-grade tumors. Survival was also strongly negatively correlated with serum MDA levels (*r* = −0.71), while a moderate positive correlation was observed with serum TAC (*r* = 0.44).

Histopathological grade demonstrated a moderate positive correlation with serum MDA concentrations (*r* = 0.45) and a weak negative correlation with serum TAC (*r* = −0.33), suggesting an association between higher tumor grade and increased systemic oxidative stress. Additionally, serum MDA showed a moderate inverse correlation with serum TAC (*r* = −0.46).

Age showed weak to moderate correlations with oxidative stress markers, including a negative correlation with serum TAC (*r* = −0.32). No strong associations were observed between spaying status and the evaluated oxidative or survival-related variables.

## 4 Discussion

Oxidative stress occurs when the production of reactive oxygen species (ROS) exceeds the cell’s ability to neutralize them. This imbalance favors the uncontrolled formation of certain covalent bonds, which can cause significant cellular damage and contribute to the development of various diseases, including cancer (24–26). In view of this, the present study evaluated the relationship between oxidative stress biomarkers and the IMC outcome in female dogs.

The biomarkers total antioxidant capacity (TAC) and malondialdehyde (MDA) are useful tools for the understanding of tumor evolution and prognosis in patients with breast cancer (6). These markers are helpful for assessing oxidative stress and interpreting disease behavior (27). In this study, canine patients with IMC had significantly higher MDA levels compared to dogs with other types of aggressive malignant mammary tumors and those with low-grade tumors. Our findings align with those of other studies, which describe an increase in this marker in both human and veterinary patients with malignant tumors (28, 29). Elevated MDA levels were reported in larger and more aggressive tumors (30), which can be explained by greater ROS production by neoplastic cells, deficiency in antioxidant mechanisms, and consequent intensification of lipid peroxidation (7, 31, 32).

The findings of the present study support a close relationship between oxidative imbalance and tumor aggressiveness in canine mammary cancer. Histopathological grade was positively correlated with serum MDA levels (*r* = 0.45) and negatively correlated with TAC (*r* = −0.33), indicating that increased lipid peroxidation and reduced antioxidant capacity are associated with a more aggressive tumor phenotype. This finding is consistent with previous studies on female dogs with malignant mammary neoplasms, in which elevated serum MDA concentrations were also associated with higher histological grades and greater biological aggressiveness (14, 15, 28).

The survival analysis showed a strong negative correlation with serum MDA concentrations (*r* = −0.71) and a moderate positive correlation with TAC (*r* = 0.44), suggesting that dogs with elevated systemic oxidative stress tend to have shorter survival. In parallel, the inverse correlation between MDA and TAC (*r* = −0.46) indicates a systemic redox imbalance, in which antioxidant defenses may be insufficient to counteract oxidative damage. Taken together, these results suggest that MDA and TAC are not only biochemical markers of oxidative status, but also could be introduced as a prognostic indicator of cancer clinical course and patient survival, particularly in inflammatory mammary carcinoma.

The association between tumor aggressiveness and notable oxidative stress is derived from the excessive metabolic demand of the tumors, due to rapid proliferation and active signaling pathways such as inflammatory signaling and oxidative phosphorylation. Following elevated tumor-related metabolism, access to required nutrients is impaired (13, 33, 34). Furthermore, metabolic reprogramming drives tumorigenesis, shifting phosphorylation toward lipid peroxidation, while the reduced antioxidant capacity favors exacerbation of the oxidative injury (11, 27, 28). This oxidative imbalance added to the elevated metabolic demands of tumor cells is associated with depletion of glucose, glutamine, cysteine, glycine, and other metabolites involved in the antioxidant defense (28). In multivariate analysis, tumor grading emerged as a significant prognostic factor associated with survival, with an Hazard Ratio (HR) of 4.79 (95% CI: 1.48–15.53; *p* = 0.009). This result indicates that, adjusted for age, TAC, and MDA, female dogs with higher tumor grading had a 4.79-fold higher hazard of death compared to those with lower grading. On the other hand, for serum levels of TAC (HR = 1.09; 95% CI: 0.81–1.47; *p* = 0.559) and MDA (HR = 1.1; 95% CI: 0.995–1.3; *p* = 0.307), multivariate analysis did not reveal a significant independent association with survival. Despite this, univariate results and marked differences in the levels of these biomarkers between tumor groups (IMC versus others) still reinforce their role in disease pathophysiology.

The strong negative correlation between survival and serum MDA reinforces lipid peroxidation as a marker of poor prognosis. Conversely, the moderate positive correlation between survival and serum TAC suggests that preserved systemic antioxidant capacity may be linked to a more favorable clinical course. Together, these associations indicate that redox imbalance is not merely a parallel phenomenon in IMC but likely reflects biological processes related to tumor progression and host systemic response.

The IMC is known for its highly metastatic capacity and low survival rate in female dogs (35). Our study showed that TAC levels were significantly lower in dogs with IMC than in dogs with either low-grade tumors or aggressive non-inflammatory tumors. This distinction suggests that IMC might induce a more pronounced redox imbalance, as evidenced by our study of dogs with this neoplasia, which showed clear low TAC and high MDA levels. This oxidative profile is consistent with the highly aggressive nature of IMC, marked by intense inflammation, rapid progression, and a poor prognosis. This evidence reinforces the role of oxidative stress not only in malignancy but also as a potential active factor in perpetuating the pro-inflammatory and pro-metastatic tumor microenvironment.

As a global indicator of antioxidant capacity, TAC plays an important role in neutralizing free radicals (36, 37). Its decrease suggests less resilience to oxidative stress and molecular damage that favors tumor progression, especially in IMC. Thus, the lower the TAC, the greater the aggressiveness and the lower the survival rate, as observed in this study.

In neoplastic cells, the increased ROS production makes them more vulnerable to oxidative damage (33). Elevated TAC levels are associated with reduced MDA-mediated lipid peroxidation (34). Furthermore, Bel’skaya and Dyachenko (11) highlighted that excessive oxidative stress favors DNA damage and malignant cell transformation, promoting tumor progression. Thus, the elevated MDA levels and reduced TAC observed in our study illustrate the participation of oxidative stress in the pathophysiology of mammary cancer in female dogs.

An additional relevant finding is that age showed only weak correlations with MDA and TAC, and spaying status showed no strong association with oxidative or survival-related variables. This suggests that, within this cohort, the oxidative profile was more strongly related to tumor biology than to baseline demographic or reproductive factors. This is clinically meaningful because it supports the potential use of MDA and TAC as disease-related biomarkers rather than merely age-dependent systemic changes.

Studies have described TAC analysis as a useful tool for predicting therapeutic response and monitoring systemic oxidative stress (38), thereby supporting the guidance of clinical strategies (39). In this context, antioxidant interventions can be useful as therapeutic tools in cancer management. Evidence in this regard was reported by Silva et al. (14), who demonstrated that ozone therapy in female dogs with mammary cancer reduced MDA levels and increased TAC during treatment, indicating that it favored redox balance by mitigating oxidative stress. In this regard, our results suggest that oxidative stress not only serves as a marker of severity but is also a promising therapeutic target.

Similarly, Oliveira et al. (15) reported that ozonated autohemotherapy resulted in substantial clinical improvement, characterized by increased TAC and a marked reduction in MDA. In that study, female dogs with mammary tumors that received ozone therapy showed better hepatorenal parameters and superior postoperative quality of life (15). In our study, no antioxidant therapeutic intervention was instituted, and TAC levels remained low in dogs with IMC, which was associated with a lower survival rate. We hypothesize that interventions aimed at modulating oxidative stress may result in a clinical impact on the quality of life and survival of dogs with severe types of mammary cancer.

The oxidative response in cancer patients is frequently linked to increased oxidative stress, accompanied by high levels of MDA in various tumor types (40). We infer that severe cell damage was possibly intensified by this high-oxidative-stress scenario in dogs with IMC, as evidenced by the low cell differentiation observed in most of the samples we analyzed, and by the greater metabolic activity associated with proliferation.

Despite the relevance of the findings, we acknowledge the limitations of the present study, including the small sample size and the lack of analysis of specific antioxidant enzymes, such as glutathione-superoxidase and catalase. The lack of correlation with data from previous cancer treatments also restricts the analysis of the therapeutic impact on oxidative stress. Further studies, with larger samples and the inclusion of enzymatic markers, are needed to expand our understanding of the mechanisms linking oxidative stress, tumor aggressiveness, and responses to various interventions.

Nevertheless, we conclude that the present results reinforce the usefulness of jointly evaluating MDA and TAC in dogs with IMC, allowing for prognostic information while helping to understand the pathophysiology of this neoplasm. Moreover, the data reinforce the importance of oxidative stress in the pathophysiology of IMC in dogs, suggesting that analyzing these biomarkers can provide valuable insights into disease progression. Therefore, the correlation profile observed in this study strengthens the interpretation that oxidative stress is linked to IMC aggressiveness and may contribute to the rapid disease progression seen in these dogs. MDA measurement presents itself as a possible prognostic marker, given its association with greater tumor aggressiveness and reduced survival. The integration of these biomarkers into clinical protocols may open new avenues for developing therapeutic strategies to modulate oxidative stress and improve clinical outcomes in canine patients with IMC.

## Data Availability

The raw data supporting the conclusions of this article will be made available by the authors, without undue reservation.
